# African Mahogany Under Saline Stress: An Analysis of the Transpiration Response at Different Salinity Levels

**DOI:** 10.3390/plants14050666

**Published:** 2025-02-21

**Authors:** Willian Viana Campos, José Teixeira Filho, Alcebíades Rebouças São José

**Affiliations:** 1 Faculty of Agricultural Engineering, Universidade Estadual de Campinas (UNICAMP), Campinas 13083871, SP, Brazil; jose@feagri.unicamp.br; 2 Plant and Animal Science Department, Southwest Bahia State University (UESB), Vitória da Conquista 45030900, BA, Brazil; alreboucas@gmail.com

**Keywords:** photosynthesis, stomatal conductance, water management, semiarid regions, water use efficiency

## Abstract

Agriculture in semi-arid regions faces significant challenges due to water scarcity and soil salinity, conditions exacerbated by inadequate irrigation practices and high evaporation rates. African mahogany (*Khaya senegalensis*), a species valued for its high-quality wood, holds potential for cultivation in these regions, provided that appropriate management practices are adopted. This study investigated the leaf transpiration response of African mahogany seedlings subjected to seven levels of irrigation water salinity, ranging from 0.5 to 5 dS·m^−1^, using drainage lysimeters in an experimental field in Bahia. Data collection included measurements of stomatal conductance and photosynthetically active radiation (Qleaf) over a four-month period. The results showed a significant reduction in transpiration with increasing salinity, particularly above 3.5 dS·m^−1^. Regression analyses highlighted a negative correlation between electrical conductivity and leaf transpiration, demonstrating the impact of water quality on plant physiology. These findings underscore the potential of African mahogany for cultivation in semi-arid regions, provided that efficient management practices are implemented to promote sustainable water use and mitigate the effects of salinity.

## 1. Introduction

Agriculture in semi-arid regions faces complex challenges due to water scarcity and adverse conditions such as high temperatures and intense solar radiation [1]. Transpiration, an essential process for plant growth, is strongly influenced by environmental factors, such as soil salinity and solar radiation [2]. This process plays a crucial role in plant water balance, directly affecting plant development, water-use efficiency, and productivity [3,4].

Soil salinity, resulting from the accumulation of soluble salts, reduces water availability to plants, compromising transpiration and, consequently, plant development. On the other hand, solar radiation, although essential for photosynthesis, influences the transpiration rate by affecting plant metabolism and physiology [5,6]. Water stress reduces daily transpiration and plant water potential [7,8], while irrigation water with high electrical conductivity further decreases water potential, hindering water uptake by plants [9]. This effect increases resistance to water penetration in plant tissues, leading to a significant drop in leaf transpiration [10].

Conditions of water stress and high salinity negatively affect plant growth and production, resulting in atrophy of conductive vessels, reduced nutrient uptake, and greater susceptibility to pathogens [11,12]. In low water potential environments, transpiration is adaptively reduced, highlighting a water conservation mechanism in plants. Solar radiation, which tends to remain stable throughout the day, interacts with water potential to influence transpiration. The lower the water potential, the weaker the transpiration response to environmental conditions.

Efficient management of African mahogany extends beyond the local context, being crucial for the international timber market, which demands sustainable agricultural practices under challenging conditions. African mahogany (*Khaya senegalensis*), valued for its high-quality wood, faces significant challenges in semi-arid regions due to soil salinity and intense solar radiation. In these regions, soil salinity is intensified by improper irrigation practices and high evaporation rates, leading to salt accumulation [13,14]. High salt levels compromise water availability to plants, causing water stress and reducing the transpiration rate [15]. Moreover, the accumulation of harmful ions such as sodium and chloride in the leaves damages the cell membranes and interferes with photosynthesis, further decreasing transpiration [16].

While numerous studies have investigated the effects of soil salinity on various crops, little attention has been given to African mahogany. Understanding its response to salinity is crucial for the proper management and conservation of water resources in regions affected by salinization. Solar radiation, in turn, is an essential factor for plant transpiration, directly influencing water evaporation from leaves and stomatal openings, which affects gas exchange [17,18].

Thus, understanding the combined effects of soil salinity and solar radiation on African mahogany transpiration is essential for sustainable agricultural practices. This study aims to provide data on the response of African mahogany to these conditions, contributing to more efficient and sustainable management strategies. The findings are expected to optimize water resource use and increase the productivity of this important crop in semi-arid regions.

## 2. Material and Methods

**Figure 1:**
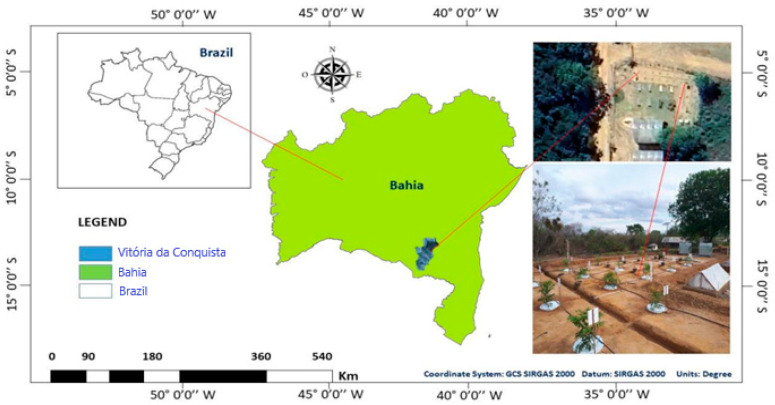
Location of the study area: Vitória da Conquista—BA, northeast of Brazil.

Lysimeters were installed in the experimental field of the State University of Southwest Bahia (UESB), located on the Vitória da Conquista campus (Figure 1). This area is located in a region characterized by a tropical high-altitude climate (Cwb), according to the Köppen classification. This indicates that the region experiences a dry season during winter, with hot and humid summers.

The geographic coordinates of the site are approximately 14°53′08″ S and 40°48′02″ W of Greenwich, with an altitude of 881 m. In the region, the rainy season occurs from November to March, with a total annual rainfall of around 700 mm. Thermal averages indicate maximum temperatures of 26.4 °C and minimum temperatures of 16.1 °C, resulting in an annual average of 20.2 °C, according to the INMET data from 2018.

### 2.1. Experimental Design

The experiment was conducted during the first nine months of crop development, with data collection on transpiration occurring in the subsequent three months: January, February, and March 2022. The evaluations were conducted over six consecutive measurement cycles, each lasting between 4 and 6 days, with daily measurements to capture the physiological responses of the plants under controlled water stress conditions. A total of 21 plants (experimental units) were evaluated, distributed across seven irrigation water salinity treatments (0.5, 1.25, 2.0, 2.75, 3.5, 4.25, and 5.0 dS·m^−1^), each with three replicates. The control treatment consisted of plants irrigated with low-salinity water (0.5 dS·m^−1^).

The plants were randomly allocated in the field, each in its own lysimeter, to avoid interference among them, and the blocks were randomized to standardize the experimental conditions. The irrigation water was prepared by adding NaCl to adjust the electrical conductivity levels, while the environmental conditions were monitored and measured daily to ensure uniformity of the treatments throughout the experiment.

### 2.2. Lysimeters

The lysimeters used were made of polyethylene (canisters), with a cylindrical shape, one meter high, and a total volume of 0.2 m^3^. They were placed at ground level, in an open area. In the center of each lysimeter, a PVC tube 75 inches in diameter and 1.6 m long was inserted. This tube was positioned so that water could be stored in it and flow to the lysimeter’s soil storage area through holes located along the tube. The part of the tube that protruded outside the lysimeter was approximately 0.6 m.

To fill the lysimeters, the following procedure was followed: an initial layer of crushed stone was added to completely cover the drainage tube holes, corresponding to a 20 cm thick layer; then, a 5 cm layer of coarse sand was placed; and finally, the inside of the lysimeter was completely filled with soil to a depth of 40 cm.

**Figure 2:**
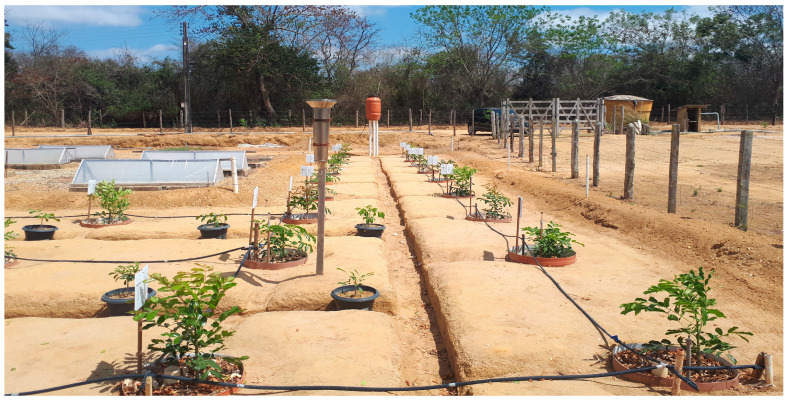
Field experiment with distribution of the drainage lysimeters in the experiment area.

After planting the African mahogany seedlings, the lysimeters were covered with a thin layer of cement to prevent water evaporation from the soil. Finally, a layer of white paint was applied to reduce the soil temperature inside the lysimeters (Figure 2).

### 2.3. Leaf Transpiration

To evaluate the transpiration (E) of the plants, a steady-state diffusion porometer, model LCpro-SD, was used. During the measurements, the plants were exposed to a certain photon irradiance (µmol·m^−2^·s^−1^ of CO_2_), according to a light saturation curve. The physiological parameters, including transpiration, were measured every 21 min. The determination of leaf transpiration was carried out at the leaf level, with individuals kept under water restriction conditions, resulting in different water potentials.

To measure the transpiration behavior, three healthy and fully expanded leaves were selected from each plant, located in the middle third of the canopy and exposed to solar radiation throughout the evaluation period. Readings were taken at hourly intervals throughout the day, from 7 a.m. to 5 p.m., for five consecutive days without applying water to the lysimeters, thus increasing water restriction to observe the effect on leaf transpiration. At the end of the five-day period, the plants were maintained under normal irrigation conditions, restoring the maximum water level in the lysimeters.

### 2.4. Leaf Water Potential (Ψw)

During the experimental period, three leaves were collected from each plant, located in the middle third of the shoot, to evaluate the leaf water potential. The leaves were collected during the morning, and the leaf water potential was determined using a pressure chamber, following the method described in [19].

### 2.5. Climate Factors

During the transpiration measurements (E), photosynthetically active radiation (Qleaf) was determined simultaneously using a sensor coupled to the porometer chamber. This sensor was positioned perpendicular to the sunlight incident on the leaf surface throughout each working day.

Furthermore, complementary data on air temperature and relative humidity, specific to the measurement days, were obtained from the meteorological station of the National Institute for Space Research (INPE). This station is located in the UESB experimental area, at a distance of 300 m from the place where the lysimeters were installed.

### 2.6. Relationship Between E, ψ, and Qleaf

With the help of SISVAR 5.6 software, regression models were developed to better explain transpiration (E) in relation to photosynthetically active radiation (Qleaf) for each class of plant water potential (ψ) at different levels of electrical conductivity.

From the models’ response curves, it was possible to determine the light saturation point for each situation, providing a more detailed understanding of the relationship between radiation and transpiration in different soil moisture and salinity conditions.

### 2.7. Analysis of Variance and Regression Analysis

The results were subjected to analysis of variance (ANOVA) using the F test to compare means and to regression analysis to quantitatively study the characteristics evaluated. These analyses were carried out using the statistical programs SISVAR 5.6 and STATISTICA 7.0. Subsequently, a regression analysis was conducted on the treatments using the Tukey test (*p* < 0.05).

For the control treatment (0.5 dSm·m^−1^), regression equations were generated using the least squares method, which sought to minimize the sum of the squares of the differences between the values estimated by the regression equation and the observed transpiration data from the mahogany crop. These differences are called residuals. To assess the quality of the model, the correlation coefficient R^2^ was used. This coefficient ranges from 0 to 1 and indicates how well the model fits the observed data, with values closer to 1 indicating a better fit of the model to the data.

R^2^ = 1 − (SQRes/SQTot)

where SQRes = sum of the squares of the residue, and SQTot = total sum of the squares.

## 3. Results

Table 1 displays the average values of the environmental parameters in the control treatment, including photosynthetically active radiation (Qleaf), leaf temperature (Tleaf), leaf transpiration (E), stomatal conductance (Gs), and photosynthesis rate (A), throughout the day for the three replicates of the control treatment. Only the values of the environmental and physiological parameters corresponding to the hours of the day when specific variations in the measurements occurred are presented. This interval was determined based on the time required to complete the measurements for all replicates, totaling 21 experimental units across the seven treatments, which took 21 min. Each measurement per experimental unit (plant) lasted one minute, resulting in 28 measurements per plant throughout the day, conducted from 7:49 a.m. to 5:59 p.m.

The values of the environmental parameters, such as photosynthetically active radiation, leaf temperature, leaf transpiration, and stomatal conductance, exhibited an oscillation pattern with greater amplitudes around midday, approximately at 12:00 p.m., and lower values at the beginning of the day, around 7:50 a.m., and at the end of the day, around 5:50 p.m. This oscillation followed a normal distribution pattern, resembling a trigonometric parabolic distribution.

In the control treatment (Table 1), the environmental parameters reached their maximum values at 12:01 p.m., with the average photosynthetically active radiation peaking at 1916.5 W·m^−2^·s^−1^, leaf temperature reaching 39.3 °C, leaf transpiration reaching 15.7 mmol·m^−2^·s^−1^, and stomatal conductance achieving its highest value of 1.5 mmol·m^−2^·s^−1^, also at 12:01 p.m. However, for photosynthesis, the oscillatory behavior did not follow a parabolic variation pattern. Instead, higher values were observed around midday, while lower values occurred at the beginning of the day, at 7:50 a.m., and at the end of the day, at 5:50 p.m. This indicated a more random data distribution throughout the day.

**Figure 3:**
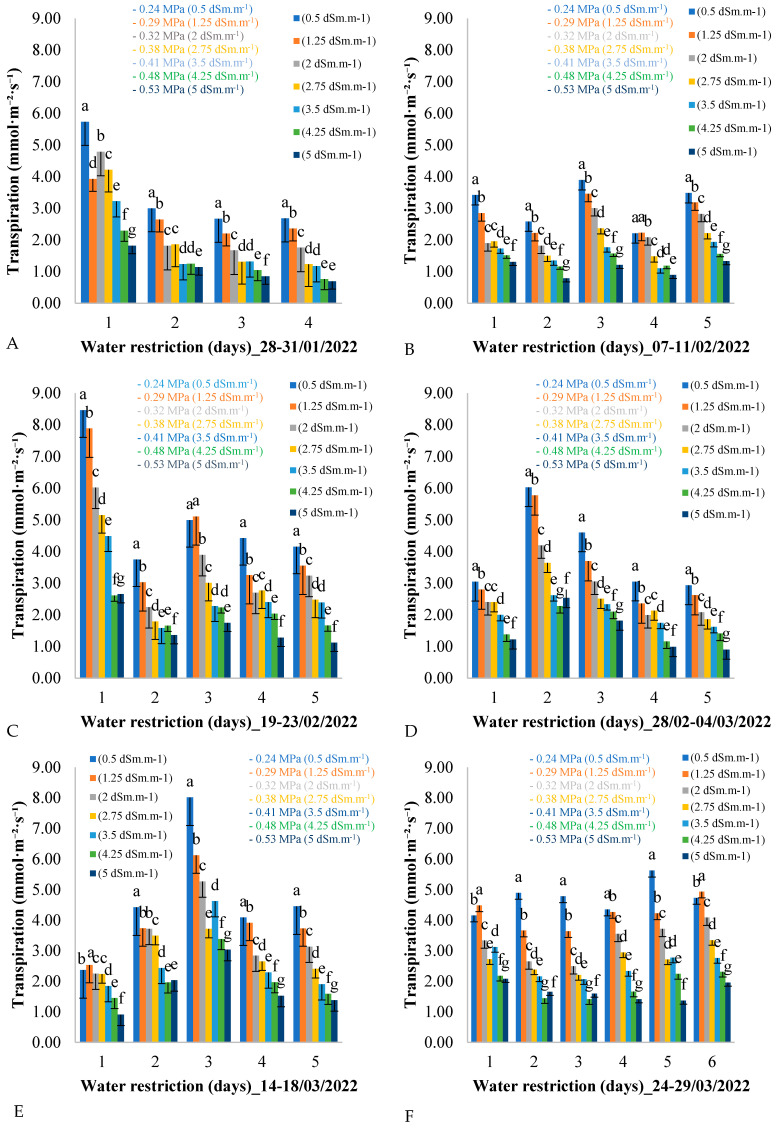
(**A**–**F**) Leaf transpiration throughout the days of the measurement cycle, from 6:00 a.m. to 6:00 p.m., in plants under different electrical conductivities and water potentials of the irrigation water for the period from 28 January to 29 March from 2022. Equal letters on bars for the same day indicate no difference between them by the Tukey test at 5% probability (*p* < 0.05).

Figure 3 shows the variation in leaf transpiration over five consecutive days for each of the six measurement cycles across the seven treatments during the field experiment assessment period. As shown in Figure 3, six evaluation periods were planned, corresponding to six measurement cycles, during which the plants were subjected to water restriction. At the end of each measurement cycle, the water supply to the African mahogany plants was restored to avoid damage caused by an excessive reduction in water potential, which could result in the death of the plants.

It can be seen in Figure 3 that, on the first day of each measurement cycle, leaf transpiration decreased as the electrical conductivity of the irrigation water increased. This pattern was repeated over the days of the measurement cycle, from day 1 to day 4, and it was also observed that the average leaf transpiration in all treatments decreased over the days of the measurement cycle during the water restriction period from 28 to 31 January 2022.

This pattern of variation in leaf transpiration was repeated during the period from 19 to 23 February 2022 (Figure 3C). For example, on the first day of the measurement cycle, leaf transpiration reached 8.5 mmol·m^−2^·day^−1^ for the control treatment of 0.5 dSm·m^−1^, while for the treatment with the highest electrical conductivity of the irrigation water (5 dSm·m^−1^), leaf transpiration was 2.5 mmol·m^−2^·day^−1^. This variation was observed on days 2, 3, 4, and 5, with the average leaf transpiration decreasing over the days of the measurement cycle with water restriction.

The variation in leaf transpiration during the period from 7 to 11 February 2022 (Figure 3B) showed similar average oscillation values between days of the measurement cycle, with variation between treatments on each day. For example, on the first day of the cycle, the treatments with lower electrical conductivity of irrigation water showed higher leaf transpiration values, while the values decreased linearly with increasing electrical conductivity.

In Figure 3D, the second day of the measurement cycle (day 2) presents the highest leaf transpiration values for all treatments. Analyzing day 2 of the period from 28 February to 4 March 2022 in isolation, the same pattern of reduction in leaf transpiration is observed between treatments, with treatments having lower electrical conductivity (0.5 dSm·m^−1^) presenting higher values (6 mmol·m^−2^·day^−1^), and treatments with higher electrical conductivity (5 dSm·m^−1^) showing lower values (2.5 mmol·m^−2^·day^−1^).

**Figure 4:**
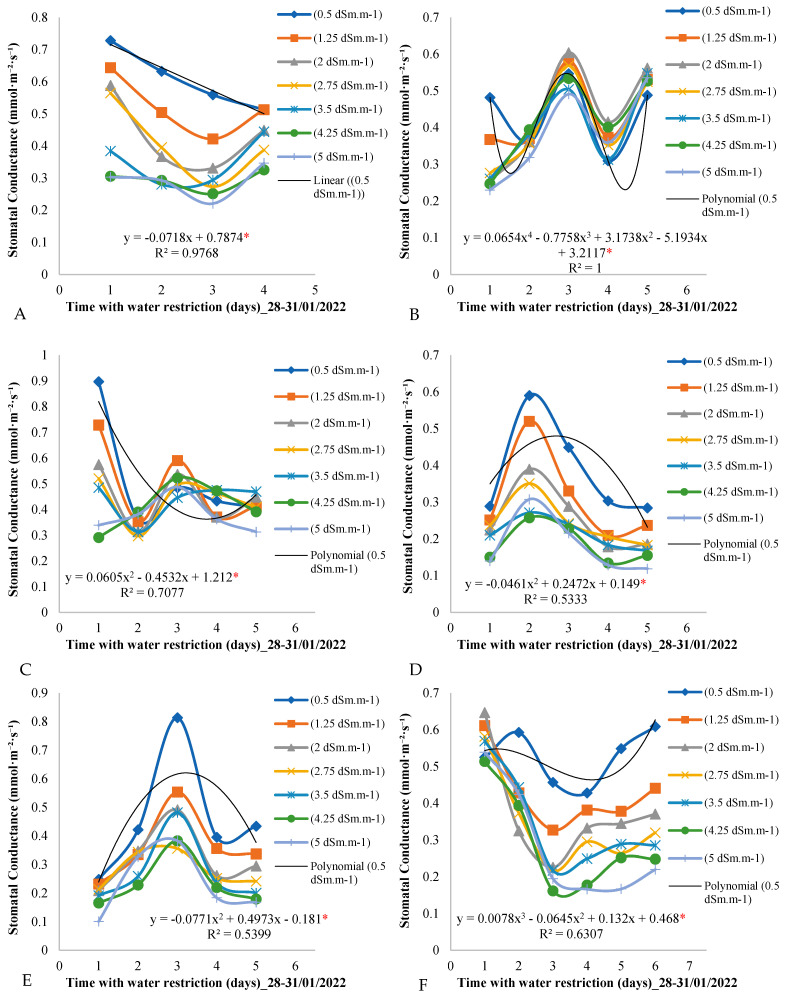
(**A**–**F**) Stomatal conductance throughout the days of the measurement cycle, from 6:00 a.m. to 6:00 p.m., in plants under different electrical conductivities of the irrigation water for the period from 28 January to 29 March from 2022. * Significant according to Tukey’s test at a 5% probability level (*p* < 0.05).

Figure 4 shows the variation in stomatal conductance over the days of the measurement cycle for the seven treatments throughout the evaluation period in the field experiment. By analyzing Figure 4A, it is observed that, on the first day of the measurement cycle (day 1) in the period from 28 to 31 January 2022, the stomatal conductance values varied according to the electrical conductivity of the irrigation water, decreasing as the conductivity increased.

This pattern of oscillation in stomatal conductance values was repeated on the second, third, and fourth days of the measurement cycle, with a gradual decrease over the days of the measurement cycle. On day 4, for example, stomatal conductance reached 0.4 mmol·m^−2^·day^−1^ for the control treatment of 0.5 dSm·m^−1^.

Figure 4B shows a variation behavior similar to that observed in the period from 28 to 31 January 2022, with a pronounced variation in the stomatal conductance values on the first day of the measurement cycle. The highest values were observed for the control treatment of 0.5 dSm·m^−1^, while they decreased with the increase in the electrical conductivity of the irrigation water.

During the period from 14 to 18 March 2022 (Figure 4C), the third day of the measurement cycle presented the highest stomatal conductance values, with the control treatment leading to a stomatal conductance of 0.8 mmol·m^−2^·day^−1^, while lower values were determined for the other treatments involving lower electrical conductivities of the irrigation water.

For the control plants subjected to electrical conductivity of the irrigation water of 0.5 dSm·m^−1^ (Figure 4F), it was observed that the stomatal conductance values were higher than those measured for the other treatments on all days of the measurement cycle. However, on the first day of the measurement cycle, these values were the lowest among those of the other treatments.

**Figure 5:**
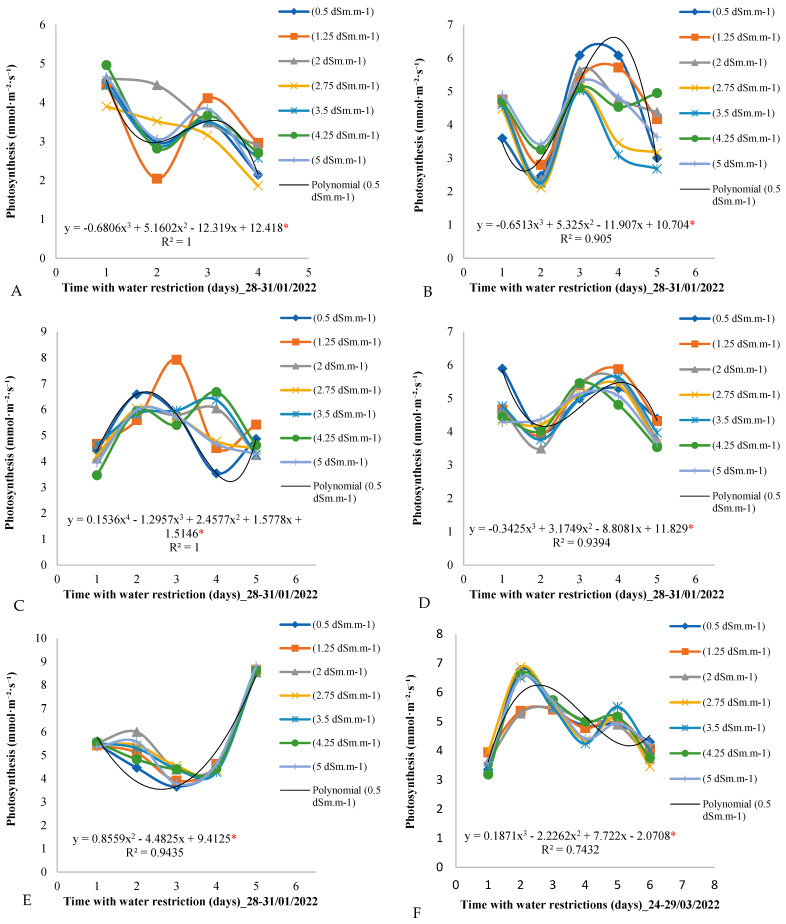
(**A**–**F**) Photosynthesis throughout the days of the measurement cycle, from 6:00 am to 6:00 pm, in plants under different electrical conductivities of the irrigation water for the period from 28 January to 29 March 2022. * Significant according to Tukey’s test at a 5% probability level (*p* < 0.05).

Figure 5 graphically presents the distribution of the photosynthetic rate over the days of the measurement cycle for all seven electrical conductivity treatments. It is observed that photosynthesis did not present a linear trend in relation to the increase in the electrical conductivity of the irrigation water. In all periods of the analysis, photosynthesis presented similar values throughout all days of the measurement cycle, not being influenced by water restriction or variations in the electrical conductivity of the irrigation water. In Figure 5E, for example, the photosynthesis values for all treatments on all days of the measurement cycle are very close, around 6 mmol·m^−2^·day^−1^, during the period from 14 to 18 March 2022.

## 4. Discussion

This study revealed clear patterns in the physiological response of African mahogany under salinity and water restriction conditions. The 21 min measurement frequency enabled a detailed analysis, highlighting notable oscillatory patterns in the values of photosynthetically active radiation (Qleaf), leaf temperature (Tleaf), leaf transpiration (E), stomatal conductance (Gs), and photosynthesis (A). Leaf transpiration significantly decreased with increasing salinity, while photosynthesis remained stable. This behavior demonstrates the species’ adaptive mechanisms to environmental stress, which are crucial for its sustainable cultivation in semi-arid regions. The oscillation in these parameters, with higher values at midday and lower values at the beginning and end of the day, is consistent with findings from other studies, indicating an adaptive response of plants to the diurnal cycle. The normal distribution pattern, resembling a trigonometric parabola, is consistent with physiological regulation to optimize metabolic efficiency.

When comparing the results with other studies, we observed that the amplitude and timing of the oscillations can vary depending on the plant species, specific environmental conditions, and experimental methods. Previous studies, as mentioned in [1,20], corroborate the general trend of midday highs, but specific divergences may occur.

There were significant peaks in photosynthetically active radiation (Table 1), reaching 1916.5 W·m^−2^·s^−1^ at 12:01. This aligns with the results of similar studies [21,22], which also identified high values during this period, indicating plant effectiveness in taking advantage of the available sunlight.

Leaf temperature reaching 42.6 °C at 2:07 p.m. indicates thermal adaptation, but this increase can influence photosynthesis and transpiration rate. Conditions of high solar radiation and temperature during the middle of the day intensified water loss through transpiration, highlighting the importance of management strategies that consider critical periods to minimize water stress. Refs. [23,24] suggest that extreme leaf temperatures can have significant effects on plant physiology, highlighting the importance of considering this factor in comparative studies.

Stomatal conductance showed a positive correlation with transpiration, confirming that higher levels of conductance are associated with greater water loss, especially in treatments with lower salinity. The more random variation observed in photosynthesis throughout the day, in contrast to a parabola pattern, suggests that this process may be more influenced by specific factors, such as the availability of water, nutrients, or other elements not considered in this study.

These results reinforce the complexity of plants’ physiological response to environmental factors, highlighting the continued need for in-depth studies to understand the nuances of these processes. This study highlighted that, despite the reduction in transpiration, photosynthesis remained stable at all salinity levels evaluated, suggesting an efficient adaptation of African mahogany to water and saline limitation.

The analysis of Figure 3 revealed interesting patterns in leaf transpiration in response to the water restriction imposed by the different treatments. The observation of the six assessment periods, each representing a measurement cycle of water restriction, provides valuable insights into how plants respond to water deficit conditions over time.

A clear trend was observed for the reduction in leaf transpiration with the increase in the electrical conductivity of the irrigation water. This pattern was consistent throughout the different periods evaluated and was observed on all days of the measurement cycle, indicating a consistent response of the plants to water availability. This inverse relationship between electrical conductivity and leaf transpiration is corroborated by previous studies [25,26,27], which highlight the importance of irrigation water quality in plant physiology.

A general decrease in leaf transpiration was also observed over the days of the measurement cycle within each evaluation period. This suggests an adaptation of the plants to water stress over time, possibly as a water conservation mechanism during periods of scarcity. This dynamic adaptation is fundamental for the survival of plants in environments subject to variations in water availability.

Transpiration varied significantly over the evaluation period. However, it is worth highlighting that the magnitude of the reduction in leaf transpiration might vary between the different treatments, as evidenced by the variation in the average values over the days of the measurement cycle. This variation can be attributed to different levels of plant tolerance to water stress, which can be influenced by genetic, environmental, and management factors.

The results in Figure 3 highlight the importance of irrigation water quality and duration of water stress in regulating plant leaf transpiration. These findings have significant implications for agricultural practices, highlighting the need for water management strategies that take into account the dynamic effects of water stress on plant physiology.

Figure 4 provides a detailed view of stomatal conductance across different treatments and time periods, providing important insights into how African mahogany plants respond to variation in the electrical conductivity of the irrigation water and the imposition of water restriction.

When observing the measurement cycle behavior of stomatal conductance, it is clear that the variation in the measured values is related to the concentration of salts in the irrigation water. In the first days of the measurement cycle (Figure 4A), a consistent decrease in stomatal conductance was noted as the electrical conductivity of the water increased. This pattern persisted in the subsequent days (Figure 4A) and reflects the physiological response of the plants to the quality of the water available for transpiration.

The average reduction in stomatal conductance throughout the measurement cycle, particularly on day 4, is an additional indication of how the plants adjusted their stomatal activity in response to water restriction. This adaptation is a known strategy of plants to conserve water during periods of drought stress [28,29,30,31,32].

Figure 4B shows a notable consistency in the pattern observed in the first time period (28–31 January 2022). The increase in electrical conductivity of the irrigation water resulted in a pronounced reduction in stomatal conductance, highlighting the sensitivity of the plants to the salt content in the water.

In the period from 19 to 23 February (Figure 4C), we observed the initial influence of water restriction on the first day of the measurement cycle, when stomatal conductance was higher in the plants under the control treatment. This suggests an immediate response by the plants to the onset of water stress, with the control plants being more resilient initially.

The change in behavior observed in Figure 4D, where the second day of the measurement cycle presents the highest stomatal conductance values, is intriguing and may indicate seasonal variation or additional external influences that deserve further investigation.

In the period from 14 to 18 March (Figure 4E), the third day of the measurement cycle stood out as the one with the highest stomatal conductance values. The control plants, once again, exhibited a higher stomatal conductance, indicating their superior capacity to respond to water stress during this period.

Figure 4F reveals that, at the end of March, the control plants maintained higher values of stomatal conductance compared to plants subjected to the other treatments, except on the first day of the measurement cycle. This persistent behavior highlights the importance of the electrical conductivity of the irrigation water in regulating stomatal conductance, especially when plants are under prolonged water stress.

An interesting finding is the variation in the magnitude of stomatal conductance between the different treatments, especially on the first day of the measurement cycle. This suggests that the plants responded differently to water availability, with treatments with lower electrical conductivity leading to higher values of stomatal conductance. This observation highlights the importance of appropriate irrigation strategies to optimize water efficiency and maximize crop productivity.

Furthermore, the identification of the second day of the measurement cycle as the period with the highest stomatal conductance values is intriguing and deserves further investigation. This observation may be related to specific environmental factors or intrinsic physiological patterns of the plants, and their complete understanding can provide valuable insights for irrigation management.

These results are consistent with previous studies, such as [33,34,35], which demonstrated the critical influence of irrigation water quality on plant stomatal physiology. Understanding these patterns is crucial to optimizing water management strategies, ensuring the health and sustainable performance of African mahogany plantations.

Figure 5 provides a visual representation of the distribution of the photosynthetic rate over the days of the measurement cycle for the different electrical conductivity values of the irrigation water. Surprisingly, the results indicated that photosynthesis did not demonstrate a clear trend of conformity towards the increasing electrical conductivity of irrigation water.

Over the six periods of analysis, photosynthesis appeared to be robust and resilient to water restriction and variation in electrical conductivity values. This suggests that for the African mahogany plants in this particular study, other factors may have been playing a more significant role in regulating the photosynthetic rate than irrigation water quality.

The lack of variation in the photosynthesis values over the days of the measurement cycle, even under different levels of water stress, indicates a possible adaptation of the plants or the ability to maintain photosynthesis within the optimal limits, regardless of the environmental conditions. This phenomenon can be attributed to complex physiological mechanisms developed by plants to optimize photosynthetic efficiency and minimize the effects of stress.

Stability in photosynthesis under different salinity levels has also been observed in species such as *Acacia senegal* and *Prosopis juliflora* [33,34], suggesting that African mahogany shares similar adaptive physiological mechanisms with these plants, which makes it ideal for sustainable cultivation in arid regions.

The period from 14 to 18 March 2022 (Figure 5E) is especially interesting, as it showed that the photosynthesis values remained consistent for all treatments on all days of the measurement cycle, with an average of around 6 mmol·m^−2^·day^−1^. This suggests that even under water stress conditions, the plants maintained a relatively stable photosynthetic rate, which is essential to ensure biomass production and healthy plant growth [36,37,38].

Evapotranspiration was observed to decrease with increasing salinity, while the leaf water potential became more negative (Figure 3), indicating a stomatal closure strategy to preserve water. This mechanism is crucial to mitigate the effects of water stress in semiarid environments.

The results indicate that irrigation at moderate salinity levels may be a viable strategy to save water without compromising photosynthesis. Techniques such as controlled irrigation and continuous monitoring of salinity are recommended to optimize the growth of African mahogany.

These results are intriguing and indicate the need for a more detailed analysis of the mechanisms underlying the regulation of photosynthesis in African mahogany plants. Future studies could explore gene expression, biochemical processes, and leaf anatomy to better understand how these plants respond to drought stress and varying environmental conditions. This information is essential for developing more effective and sustainable management strategies for African mahogany plantations.

## 5. Conclusions

The study demonstrated that African mahogany (*Khaya senegalensis*) exhibits significant adaptive capacity under moderate salinity conditions, maintaining stable photosynthesis under electrical conductivity levels up to 3.5 dS·m^−1^. This behavior suggests that the use of irrigation water with moderate salinity is a viable practice in semi-arid regions, contributing to water sustainability without compromising plant growth. However, levels above 3.5 dS·m^−1^ resulted in a marked reduction in transpiration, which may limit long-term plant development, emphasizing the need for continuous water quality monitoring. The results suggest that African mahogany possesses robust physiological mechanisms, such as mechanisms ensuring stability in photosynthesis, even under drought stress. Future studies may focus on biochemical markers, such as malondialdehyde, catalase, and peroxidase, to elucidate the antioxidant mechanisms that contribute to this tolerance. The environmental measurements showed predictable diurnal fluctuations, with peaks in light intensity and temperature at midday, while leaf transpiration and stomatal conductance progressively decreased with increasing salinity. Photosynthesis, on the other hand, remained stable, indicating robust physiological mechanisms that enable the species to tolerate water and saline stress conditions. These results highlight the potential of African mahogany for cultivation in semi-arid regions and reinforce the importance of management strategies that include the use of moderate saline water and efficient irrigation practices. Future research should explore the underlying mechanisms of this tolerance to optimize management and ensure the species’ productive sustainability.

## Figures and Tables

**Table 1:** Environmental parameters for control treatment (3 January 2022), including photosynthetically active radiation (Qleaf), leaf temperature (Tleaf), leaf transpiration (E), stomatal conductance (Gs), and photosynthesis (A), throughout the day, with mean data (*n* = 3). * Standard Error.

Time (hs:m)	Qleaf (W·m^−2^·s^−1^)	Tleaf (°C)	E (mmol·m^−2^·s^−1^)	Gs (mmol·m^−2^·s^−1^)	A (mmol·m^−2^·s^−1^)
07:49	66 (* SE = ±0.1)	23.2 (SE = ±0.1)	0.4 (SE = ±0.1)	0.1 (SE = ±0.1)	3.6 (SE = ±0.1)
09:13	1320.5 (SE = ±1.1)	28.9 (SE = ±0.1)	10.8 (SE = ±0.1)	1.1 (SE = ±0.1)	4.4 (SE = ±0.1)
12:01	1916.5 (SE = ±1.1)	39.3 (SE = ±0.1)	15.7 (SE = ±0.1)	1.5 (SE = ±0.1)	5.1 (SE = ±0.1)
14:07	1302 (SE = ±1.0)	42.6 (SE = ±0.1)	10.6 (SE = ±0.1)	1 (SE = ±0.1)	2.4 (SE = ±0.1)
16:33	220.5 (SE = ±1.0)	34.2 (SE = ±0.1)	1.8 (SE = ±0.1)	0.2 (SE = ±0.1)	3.4 (SE = ±0.1)
17:57	24.5 (SE = ±0.1)	29.1 (SE = ±0.1)	0.2 (SE = ±0.1)	0 (SE = ±0.1)	2.9 (SE = ±0.1)

## Data Availability

The data presented in this study are available in: https//doi.org/10.17632/78gk7rng6w.1, Viana Campos, Willian (2025), “African Mahogany Work Research Dataset Under Salt Stress”, Mendeley Data, V1.
